# Supplementary material for: Fungal Whole-Genome Sequencing for Species Identification: From Test Development to Clinical Utilization
Source: J Fungi (Basel). 2023 Jan 29;9(2):183. doi: 10.3390/jof9020183 (PMC9965959; doi:10.3390/jof9020183)

Supplemental Table S1. List of fungal isolates and related information.

Study ID	Organism Type	Original ID	Source	Method Used for Original ID	WGS ID	Marker/Method Used for WGS ID
UCLA_024	Mold - Dematiaceous	*Scedosporium apiospermum*	Eye, Cornea	Microscopic morphology	*Scedosporium dehoogii*	ITS
UCLA_058	Mold - Mucorales	*Lichtheimia ornata*	N/A	DNA Sequencing - UT San Antonio	*Lichtheimia ornata*	28S
UCLA_076	Mold - Mucorales	*Rhizomucor pusillus*	N/A	DNA Sequencing - UT San Antonio	*Rhizomucor pusillus*	28S
UCLA_144	Mold - Dematiaceous	*Scedosporium apiospermum*	Eye, Cornea	Microscopic morphology	*Scedosporium dehoogii*	ITS
UCLA_145	Yeast	*Candida auris*	Swab, Inguinal/Axillary	MALDI-TOF MS	*Candida auris*	ITS
UCLA_146	Mold - Dimorphic	*Blastomyces dermatitidis*	Bronchoalveolar Lavage	Accuprobe	*Blastomyces dermatitidis*	ITS
UCLA_147	Yeast	*Candida auris*	N/A	Reference Isolate	*Candida auris*	ITS
UCLA_148	Mold - Mucorales	*Syncephalastrum spp.*	Tissue, Abdomen	Microscopic morphology	*Syncephalastrum racemosum*	28S
UCLA_149	Yeast	*Cryptococcus neoformans*	N/A	Reference Isolate (ATCC 32045)	*Cryptococcus neoformans*	ITS
UCLA_150	Mold - Hyaline	*Fusarium proliferatum*	Tracheal Aspirate	DNA Sequencing - UT San Antonio	*Fusarium proliferatum*	ITS
UCLA_151	Mold - Hyaline	*Paecilomyces variotti*	Endotracheal	Microscopic morphology	*Paecilomyces variotii*	ITS
UCLA_154	Yeast	*Candida auris*	Tracheal Suction	MALDI-TOF MS	*Candida auris*	ITS
UCLA_156	Yeast	*Candida haemulonii*	N/A	Reference Isolate (CDC AR0393)	*Candida spp.* most closely related to *C. haemulonii*	ITS
UCLA_158	Mold - Dematiaceous	*Scedosporium prolificans*	Bronchoalveolar Lavage	Microscopic morphology	*Scedosporium prolificans*	ITS
UCLA_160	Mold - Hyaline	*Aspergillus terreus*	Tracheal Aspirate	Microscopic morphology	*Aspergillus terreus*	K-mer Analysis & beta-tubulin
UCLA_164	Dermatophyte	*Trichophyton tonsurans*	Skin Scraping	Microscopic morphology	*Trichophyton tonsurans*	ITS
UCLA_261	Mold - Hyaline	*Aspergillus niger*	Bronchoalveolar Lavage	Microscopic morphology	*Aspergillus tubingensis*	K-mer Analysis & beta-tubulin
UCLA_262	Mold - Hyaline	*Aspergillus niger*	Tracheal Aspirate	Microscopic morphology	*Aspergillus niger*	K-mer Analysis & beta-tubulin
UCLA_274	Mold - Hyaline	*Aspergillus nidulans*	Expectorated Sputum	Microscopic morphology	*Aspergillus quadrilineatus*	K-mer Analysis & beta-tubulin
UCLA_273	Mold - Dermatophyte	*Trichophyton mentagrophytes*	Nails, Toe	Microscopic morphology	*Trichophyton mentagrophytes*	ITS
UCLA_272	Mold - Dermatophyte	*Trichophyton rubrum*	Biopsy, Leg	Microscopic morphology	*Trichophyton rubrum*	ITS
UCLA_280	Mold - Hyaline	*Aspergillus terreus*	Expectorated Sputum	Microscopic morphology	*Aspergillus terreus*	K-mer Analysis & beta-tubulin
UCLA_281	Mold - Hyaline	*Aspergillus fumigatus*	Swab, Maxillary Sinus	Microscopic morphology	*Aspergillus fumigatus*	K-mer Analysis & beta-tubulin
UCLA_283	Mold - Mucorales	*Syncephalastrum spp.*	N/A	Reference Isolate	*Syncephalastrum monosporum*	28S
UCLA_287	Mold - Dematiaceous	*Alternaria spp.*	Nails, Toe	MALDI-TOF MS	*Alternaria alternata/cerealis/alstroemeriae/gossypina/tenuissima*	ITS
UCLA_289	Mold - Hyaline	*Aspergillus fumigatus*	Pleural Fluid	Microscopic morphology	*Aspergillus fumigatus*	K-mer Analysis & beta-tubulin

UCLA_291	Mushroom-forming Fungi	*Coprinellus micaceus*	Bronchoalveolar lavage	MALDI-TOF MS	*Coprinellus spp.* most closely related to *C. micaceus*	ITS
UCLA_292	Mold - Hyaline	*Fusarium spp.*	Blood	Microscopic morphology	*Fusarium dimerum*	ITS
UCLA_293	Mold - Dimorphic	*Coccidioides immitis*	CSF	Accuprobe	*Coccidioides immitis*	ITS
UCLA_294	Mold - Dimorphic	*Coccidioides immitis*	Expectorated Sputum	Accuprobe	*Coccidioides immitis*	ITS
UCLA_295	Mold - Dimorphic	*Coccidioides immitis*	Synovial Fluid	Accuprobe	*Coccidioides immitis*	ITS
UCLA_296	Mold - Hyaline	*Scopulariopsis brevicaulis*	Swab, Sinus	Microscopic morphology	*Scopulariopsis brevicaulis*	ITS
UCLA_297	Mold - Hyaline	*Aspergillus sydowii*	Nails, Finger	Microscopic morphology	*Aspergillus sydowii*	K-mer Analysis & beta-tubulin
UCLA_299	Mold - Dimorphic	*Sporothrix schenckii*	Synovial Fluid	Microscopic morphology	*Sporothrix schenckii*	ITS
UCLA_300	Mold - Dematiaceous	*Bipolaris spp.*	Induced Sputum	Microscopic morphology	*Bipolaris spicifera*	ITS
UCLA_301	Mold - Dematiaceous	*Exophiala dermatitidis*	Bronchoalveolar lavage	Microscopic morphology	*Exophiala dermatitidis*	ITS
UCLA_302	Mold - Mucorales	*Mucor spp.*	Tissue, Thigh	Microscopic morphology	*Mucor circinelloides*	28S
UCLA_303	Mold - Mucorales	*Rhizopus spp.*	Swab, Bronchus	Microscopic morphology	*Rhizopus microsporus*	28S
UCLA_305	Mold - Hyaline	*Aspergillus flavus-oryzae*	Exudate, Earlobe	Microscopic morphology	*Aspergillus flavus*	K-mer Analysis & beta-tubulin
UCLA_306	Mold - Hyaline	*Aspergillus ustus*	N/A	Reference Isolate	*Aspergillus calidoustus*	K-mer Analysis & beta-tubulin
UCLA_298	Mold - Dematiaceous	*Exophiala dermatitidis*	Expectorated Sputum	Microscopic morphology	*Exophiala dermatitidis*	ITS
UCLA_304	Mold - Hyaline	*Myrmecridium schulzeri*	Endotracheal	Microscopic morphology	*Myrmecridium schulzeri*	ITS
UCLA_307	Mold - Mucorales	*Circinella spp.*	N/A	DNA Sequencing - UT San Antonio	*Circinella muscae*	28S
UCLA_308	Mold - Dermatophyte	*Neoscytalidium dimidiatum*	N/A	Reference Isolate	*Neoscytalidium dimidiatum*	ITS
UCLA_309	Mold - Mucorales	*Mucor spp.*	Bone, Foot	Microscopic morphology	*Mucor circinelloides*	28S
UCLA_310	Mold - Mucorales	*Rhizopus spp.*	Bronchoalveolar lavage	Microscopic morphology	*Rhizopus arrhizus* (*orzyae*)	28S
UCLA_311	Mold - Dimorphic	*Sporothrix schenckii*	N/A	Reference Isolate	N/A	N/A
UCLA_312	Mold - Hyaline	*Aspergillus flavus-oryzae*	Bronchial Wash	Microscopic morphology	*Aspergillus flavus*	K-mer Analysis & beta-tubulin
UCLA_316	Mold - Dermatophyte	*Epidermophyton floccosum*	N/A	Reference Isolate	*Epidermophyton floccosum*	ITS
UCLA_317	Mold - Dermatophyte	*Microsporum audouinii*	N/A	Reference Isolate	*Microsporum audouinii*	ITS
UCLA_318	Mold - Dermatophyte	*Microsporum canis*	N/A	Reference Isolate	*Microsporum canis*	ITS
UCLA_319	Mold - Dermatophyte	*Microsporum gypseum*	N/A	Reference Isolate	*Microsporum gypseum*	ITS
UCLA_320	Yeast	*Blastobotrys raffinosifermentans*	Swab, Sternum	DNA Sequencing - UT San Antonio	*Blastobotrys raffinosifermentans*	ITS
UCLA_321	Yeast	*Blastobotrys raffinosifermentans*	Pleural Fluid	DNA Sequencing - UT San Antonio	*Blastobotrys raffinosifermentans*	ITS
UCLA_371	Yeast	*Rhodotorulla spp.*	N/A	Reference Isolate	*Rhodotorula mucilaginosa*	ITS
UCLA_372	Yeast	*Candida kefyr*	N/A	Reference Isolate (CDC AR0588)	*Candida kefyr*	ITS

UCLA_373	Yeast	*Candida guilliermondii*	N/A	Reference Isolate (CDC AR0590)	*Candida guilliermondii*	ITS
UCLA_374	Yeast	*Candida tropicalis*	N/A	Reference Isolate (CDC AR3045)	*Candida tropicalis*	ITS
UCLA_378	Yeast	*Candida duobushaemulonii*	N/A	Reference Isolate (CDC AR0391)	*Candida duobushaemulonii*	ITS
UCLA_379	Yeast	*Saccharomyces cerevisiae*	N/A	Reference Isolate (CDC AR0399)	*Saccharomyces cerevisiae*	ITS
UCLA_380	Yeast	*Candida parapsilosis*	N/A	Reference Isolate (CDC AR0339)	*Candida parapsilosis*	ITS
UCLA_415	Yeast	*Cryptococcus albidus*	Blood	API	*Cryptococcus liquefaciens*	ITS
UCLA_417	Mold - Hyaline	*Aspergillus terreus*	Bronchoalveolar Lavage	Microscopic morphology	*Aspergillus terreus*	K-mer Analysis & beta-tubulin
UCLA_423	Yeast	*Candida fermentati*	Induced Sputum	MALDI-TOF MS	*Candida fermentati*	ITS
UCLA_424	Yeast	*Candida spp.*	Blood	MALDI-TOF MS	*Candida dubliniensis*	ITS
UCLA_425	Yeast	*Candida spp.*	Blood	MALDI-TOF MS	*Candida dubliniensis*	ITS
UCLA_429	Yeast	*Candida fermentati*	Induced Sputum	MALDI-TOF MS	*Candida fermentati*	ITS
UCLA_430	Yeast	*Candida fermentati*	Nails, Finger	MALDI-TOF MS	*Candida fermentati*	ITS
UCLA_530	Yeast	*Cryptococcus gattii*	N/A	Reference Isolate (ATCC 4560)	*Cryptococcus gattii*	ITS
UCLA_531	Yeast	*Cryptococcus laurentii*	N/A	Reference Isolate (ATCC 18803)	*Cryptococcus laurentii*	ITS
UCLA_536	Mold - Hyaline	*Aspergillus fumigatus*	Wound, Chest	Microscopic morphology	*Aspergillus fumigatus*	K-mer Analysis & beta-tubulin
UCLA_581	Mold - Hyaline	*Penicillium spp.*	N/A	Reference Isolate	*Penicillium solitum*	ITS
UCLA_582	Mold - Hyaline	*Trichoderma spp.*	N/A	Reference Isolate	*Trichoderma harzianum/lixii*	ITS
UCLA_583	Mold - Dematiaceous	*Curvularia spp.*	N/A	Reference Isolate	*Curvularia lunata/hominis/platzii*	ITS
UCLA_600	Mold - Dimorphic	*Histoplasma capsulatum*	Tissue, Lymph Node	MALDI-TOF MS	*Histoplasma capsulatum*	ITS

N/A=Not Applicable, CSF=Cerebrospinal Fluid

Supplemental Table S2. Impact of fungal isolate pre-treatment methods on organism viability.

Organism	Heat (100°C, 15min) + bead beat	Heat (100°C, 30min) + bead beat	70% EtOH + bead beat	70% EtOH + Heat (100°C, 30min) + bead beat
Aspergillus brasiliensis	Growth	Growth	No growth	No growth
Blastomyces dermatitidis	Growth	No growth	N/A	No growth
Scedosporium apiospermum	No growth	No growth	N/A	No growth
Microsporum gypseum	No growth	No growth	N/A	No growth
Exophiala dermatitidis	No growth	No growth	N/A	No growth
Fusarium proliferatum	No growth	No growth	N/A	No growth
Candida auris	No growth	No growth	N/A	No growth
Cryptococcus neoformans	No growth	No growth	N/A	No growth
Coccidioides immitis	No growth	No growth	N/A	No growth
Candida haemulonii	No growth	No growth	N/A	No growth
Kodamaeae ohmeri	No growth	No growth	N/A	No growth
Candida lusitaniae	No growth	No growth	N/A	No growth
Acremonium spp.	No growth	No growth	N/A	No growth
Aspergillus terreus	No growth	No growth	N/A	No growth
Candida albicans	No growth	No growth	N/A	No growth
Scedosporium prolificans	No growth	No growth	N/A	No growth
Trichophyton tonsurans	No growth	No growth	N/A	No growth
Cryptococcus gattii	No growth	No growth	N/A	No growth
Rhizopus spp.	No growth	No growth	N/A	No growth

EtOH=Ethanol, N/A=Not Applicable
Organisms incubated for 4 weeks before determined to be “No growth”, except for *B. dermatitidis* which was incubated for 6 weeks.

Supplemental Table S3. Sequencing run QC metrics and results.

Metric	Passing Criteria	Comment	Average (SD)	Pass Rate (%)
% PF	≥60%	Mandatory criteria	97 (3)	100% (20/20)
Evenness of % PF	≥2% per sample	Mandatory criteria	8 (5)	99% (74/75)[a]
Calculated Total Reads	1,000,000	Mandatory criteria	3027986 (1720534)	99% (74/75)[a]
% Undetermined Reads	<5%	Optimal criteria	2 (0)	100% (20/20)
Lane Density	1000±400	Optimal criteria	980 (217)	90% (18/20)
% >Q30 (Read 1)	≥60%	Mandatory criteria	90 (3)	100% (20/20)
% >Q30 (Read 2)	≥60%	Mandatory criteria	95 (4)	100% (20/20)
% >Q30 (Read 3)	≥60%	Mandatory criteria	94 (4)	100% (20/20)
% >Q30 (Read 4)	≥60%	Mandatory criteria	80 (5)	100% (20/20)

[a] Isolate UCLA_311 (*Sporothrix schenckii*) was excluded from the subsequent analyses because the % PF per sample was <2% (0.77%); the total reads were 277,700.

SD=Standard Deviation

PF=Passing Filter

Supplemental Table S4. Isolate QC metrics and results.

Metric	Range	Average (SD)	QC Criteria
***de novo* Assembly:**			
N50	1,446-1,191,171	141,681 (225,637)	None
GC Content (%)	33.00-60.50	47.54 (5.49)	None
**ITS Mapping:**			
Average Coverage	204-4622	982 (684)	None
5X Coverage (%)	66.16-100	98.67 (4.68)	≥80%
10X Coverage (%)	66.16-100	98.41 (5.19)	≥80%
**28S Mapping (Mucorales):**	N=9	N=9	
Average Coverage	448-2179	1098 (620)	None
5X Coverage (%)	99.43-100	99.81 (0.25)	≥80%
10X Coverage (%)	98.73-100	99.71 (0.44)	≥80%

SD=Standard Deviation

Supplemental Table S5. In-house *Aspergillus* Reference Genomes

Species	Strain Identifier
Aspergillus aculeatus	ATCC16872
Aspergillus alliaceus	Aspalli1
Aspergillus amoenus	ASM981243v1
Aspergillus arachidicola	Aspara19utr
Aspergillus avenaceus	Aspave1
Aspergillus awamori	Aawm
Aspergillus bertholleticus	Aspber1
Aspergillus bombycis	ASM179269
Aspergillus brasiliensis	CBS101740
Aspergillus brunneoviolaceus	Aspbru1
Aspergillus caelatus	Aspcae1
Aspergillus calidoustus	Acal_Allpaths_LG
Aspergillus campestris	IBT28561
Aspergillus candidus	Aspcand1
Aspergillus carbonarius	ITEM5010
Aspergillus cejpii	ASM476916
Aspergillus chevalieri	JCM_23047
Aspergillus clavatus	NRRL1
Aspergillus coremiiformis	Aspcor1
Aspergillus costaricaensis	Aspcos1
Aspergillus cristatus	ASM169335
Aspergillus ellipticus	Aspell1
Aspergillus eucalypticola	Aspeuc1
Aspergillus fijiensis	Aspfij1
Aspergillus fischer	iNRRL181
Aspergillus flavus	NRRL3357
Aspergillus fumigatus	A1163

Aspergillus fumigatus	Af293
Aspergillus glaucus	CBS516.65
Aspergillus hancockii	ASM169659
Aspergillus heteromorphus	Asphet1
Aspergillus homomorphus	Asphom1
Aspergillus ibericus	Aspibe1
Aspergillus indologenus	Aspind1
Aspergillus japonicus	Aspjap1
Aspergillus kawachii	IFO4308
Aspergillus lentulus	Alt_assembly01
Aspergillus leporis	Asplep1
Aspergillus luchuensis	CBS106.47
Aspergillus minisclerotigenes	Aspmin1
Aspergillus mulundensis	ASM336962
Aspergillus neoellipticus	ASM311656
Aspergillus neoniger	Aspneo1
Aspergillus nidulans	FGSCA4
Aspergillus niger	ATCC1015
Aspergillus niger	ATCC13496
Aspergillus niger	CBS513-88
Aspergillus niger	N402ATCC64974
Aspergillus nomius	ASM120477v1
Aspergillus novofumigatus	IBT16806
Aspergillus novoparasiticus	Aspnovo1
Aspergillus ochraceoroseus	IBT24754
Aspergillus ochraceus	AoFc
Aspergillus olivimuriae	ASM371941
Aspergillus oryzae	RIB40
Aspergillus parasiticus	Asppar1

Aspergillus persii	ASM221596
Aspergillus piperis	Asppip1
Aspergillus pseudocaelatus	Asppsec1
Aspergillus pseudonomius	Asppsen1
Aspergillus pseudotamarii	Asppset1
Aspergillus pseudoterreus	ASM292700
Aspergillus rambellii	ASM98664
Aspergillus ruber	Eurhe1
Aspergillus saccharolyticus	Aspsac1
Aspergillus sclerotialis	phiScl
Aspergillus sclerotiicarbonarius	Aspscle1
Aspergillus sclerotioniger	Aspscl1
Aspergillus sclerotiorum	HBR18v1
Aspergillus sergii	Aspser1
Aspergillus sojae	ASM827498v1
Aspergillus spinulosporus	ASM357481
Aspergillus steynii	IBT23096
Aspergillus sydowii	CBS593.65
Aspergillus taichungensis	Asptaic1
Aspergillus tamarii	Asptam1
Aspergillus tanneri	ASM342696
Aspergillus terreus	NIH2624
Aspergillus thermomutatus	ASM223726
Aspergillus transmontanensis	Asptra1
Aspergillus tritici	ASM981242v1
Aspergillus tubingensis	CBS134.48
Aspergillus turcosus	ASM223496
Aspergillus udagawae	Aud_assembly01
Aspergillus unguis	ASM332417

Aspergillus ustus	Austus1.0
Aspergillus uvarum	Aspuva1
Aspergillus vadensis	Aspvad1
Aspergillus versicolor	CBS583.65
Aspergillus violaceofuscus	Aspvio1
Aspergillus viridinutans	ASM436809
Aspergillus welwitschiae	Aspwel1
Aspergillus wentii	DTO134E9
Aspergillus westerdijkiae	ASM130734
Aspergillus zonata	CBS506.65
Aspergillus quadrilineatus	ASM1330552v1

Supplemental Table S6. Precision results.

	WGS ID	ITS Region Query	
		% Overlap	% Similarity
**Precision (between run):**			
***C. immitis* (Mold_EXT_01)**			
Rep1	*Coccidioides immitis*	100	100
Rep2	*Coccidioides immitis*	100	100
Rep3	*Coccidioides immitis*	100	99.82
***C. tropicalis* (Yeast_EXT_01)**			
Rep1	*Candida tropicalis*	100	100
Rep2	*Candida tropicalis*	100	100
Rep3	*Candida tropicalis*	100	100
**Precision (within run):**			
C. immitis			
Mold_EXT_01	*Coccidioides immitis*	100	100
Mold_EXT_02	*Coccidioides immitis*	99.81	100
Mold_EXT_03	*Coccidioides immitis*	100	100
C. tropicalis			
Yeast_EXT_01	*Candida tropicalis*	100	100
Yeast_EXT_02	*Candida tropicalis*	100	100
Yeast_EXT_03	*Candida tropicalis*	100	100

Strains used: *Coccidioides immitis* UCLA_293, *Candida tropicalis* UCLA_374
Mold_EXT_01, 02, and 03 are three unique preparations (sub-culture and DNA extraction) of *C. immitis*
Yeast_EXT_01, 02, and 03 are three unique preparations (sub-culture and DNA extraction) of *C. tropicalis*

Supplemental Table S7. *In silico* validation of the Westerdijk Fungal Biodiversity Institute database using reference ITS sequences.

	Organism	NCBI Accession	WGS ID	Query Result: %Overlap/%Similarity	
				ITS	28S
VAL_01	*Bipolaris victoriae*	NR_147489	*Bipolaris victoriae*	100/100	N/A
VAL_02	*Curvularia lunata*	NR_138223	*Curvularia lunata*	100/100	N/A
VAL_03	*Exserohilum turcicum*	NR_163537	*Exserohilum turcicum*	100/100	N/A
VAL_04	*Trichophyton rubrum*	NR_131330	*Trichophyton rubrum*	100/100	N/A
VAL_05	*Microsporum audouinii*	NR_144883	*Microsporum audouinii*	100/100	N/A
VAL_06	*Epidermophyton floccosum*	NR_131275	*Epidermophyton floccosum*	100/100	N/A
VAL_07	*Blastomyces dermatitidis*	NR_163503	*Blastomyces dermatitidis*	100/100	N/A
VAL_08	*Histoplasma capsulatum*	NR_149341	*Histoplasma capsulatum*	99.62/100	N/A
VAL_09	*Coccidioides immitis*	NR_157446	*Coccidioides immitis*	100/100	N/A
VAL_10	*Aspergillus nidulans*	NR_133684	*Aspergillus spp.*	100/100	N/A
VAL_11	*Aspergillus fumigatus*	NR_121481	*Aspergillus spp.*	100/100	N/A
VAL_12	*Aspergillus flavus*	NR_111041	*Aspergillus spp.*	100/100	N/A
VAL_13	*Fusarium fujikuroi*	NR_111889	*Fusarium fujikuroi*	100/100	N/A
VAL_14	*Beauveria bassiana*	NR_111594	*Beauveria bassiana*	100/100	N/A
VAL_15	*Mucor indicus*	NG_057878	*Mucor indicus*	N/A	100/99.86
VAL_16	*Rhizopus oryzae*	NG_056282	*Rhizopus oryzae*	N/A	100/100
VAL_17	*Lichtheimia ramosa*	NG_042518	*Lichtheimia ramosa*	N/A	100/100
VAL_18	*Candida tropicalis*	NR_111250	*Candida tropicalis*	100/100	N/A
VAL_19	*Candida albicans*	NR_125332	*Candida albicans*	100/100	N/A
VAL_20	*Candida auris*	NR_154998	*Candida auris*	100/100	N/A
VAL_21	*Cryptococcus gattii*	NR_165941	*Cryptococcus gattii*	100/100	N/A
VAL_22	*Trichosporon coremiiforme*	NR_073249	*Trichosporon coremiiforme*	100/100	N/A

N/A=Not Applicable

Supplemental Figure S1. CLCbio Workflow for Fungal Species Identification

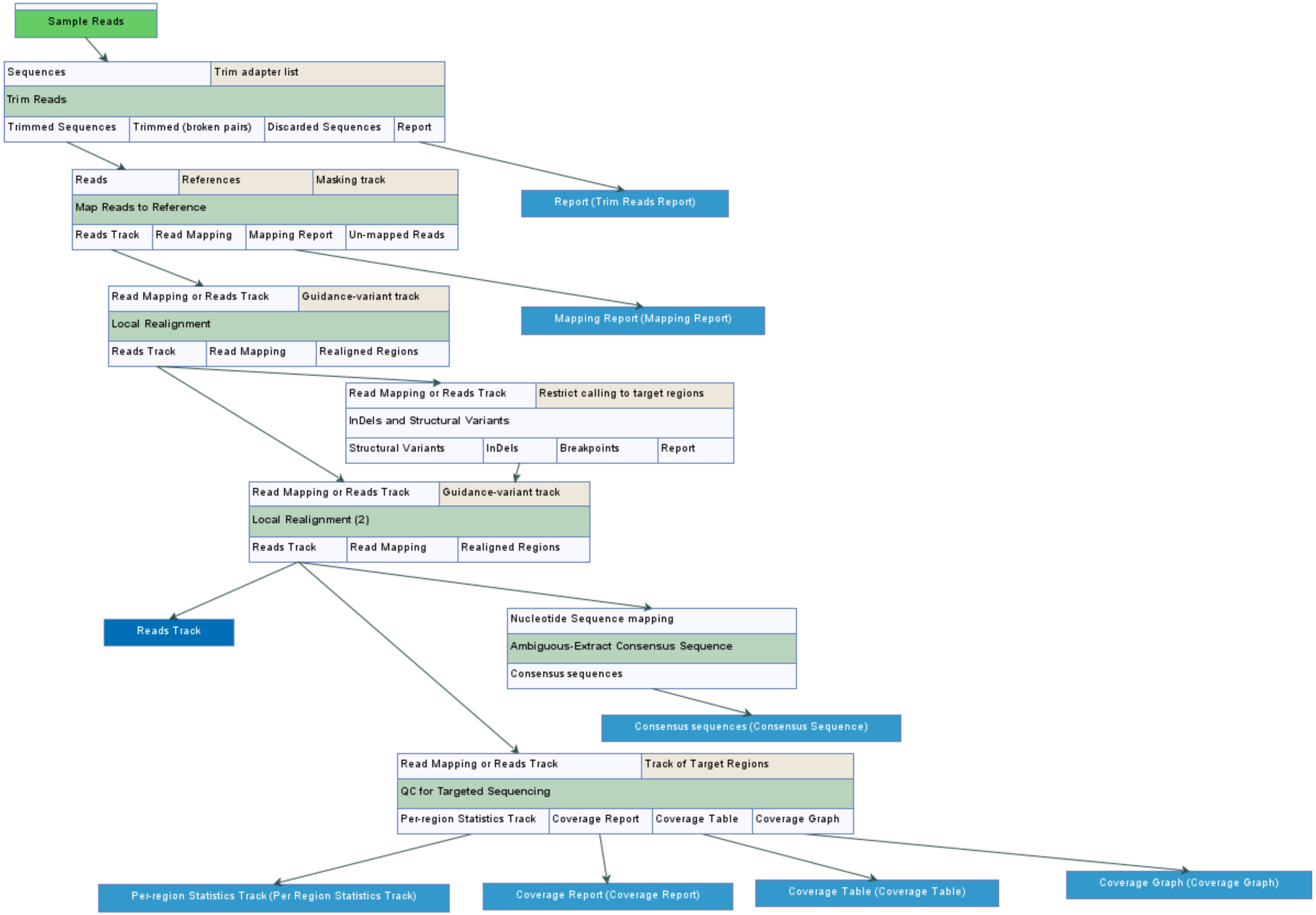

Supplemental Figure S2. Examples of K-mer Tree Analysis for *Aspergillus*

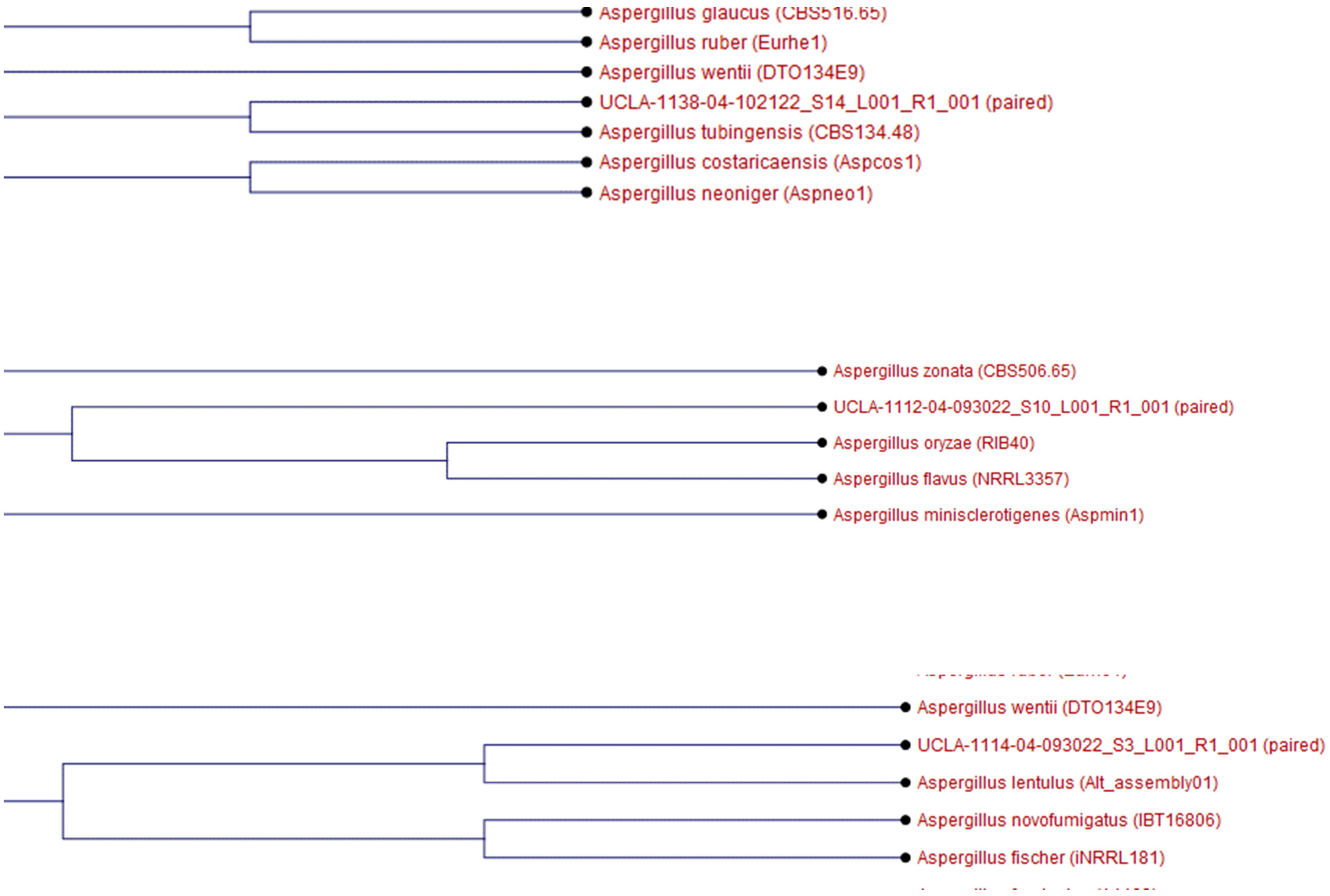

Supplement: Supplementary file 1 [file jof-09-00183-s001.zip › jof-2173133-supplementary.pdf]
